# Exploration of lncRNA/circRNA-miRNA-mRNA network in patients with chronic atrophic gastritis in Tibetan plateau areas based on DNBSEQ-G99 RNA sequencing

**DOI:** 10.1038/s41598-024-59836-4

**Published:** 2024-04-22

**Authors:** Wen Pan, Chao Liu, Tao Ren, Xiaohong Chen, Cuiting Liang, Jin Wang, Jinlin Yang

**Affiliations:** 1 Department of Gastroenterology and Hepatology, West China Hospital of Sichuan University, 37 Guoxue Lane, Wuhou District, Chengdu, 610054 Sichuan China; 2 Department of Health Management Center, The Hospital of Chengdu Office of People’s Government of Tibetan Autonomous Region, Chengdu, Sichuan China; 3 Department of Gastroenterology and Hepatology, The Hospital of Chengdu Office of People’s Government of Tibetan Autonomous Region, Chengdu, Sichuan China

**Keywords:** Chronic atrophic gastritis, Tibetan plateau areas, RNA sequencing, circRNA-miRNA-mRNA, lncRNA-miRNA-mRNA, Gastroenteritis, Genomics

## Abstract

A higher incidence of chronic atrophic gastritis (CAG) is generally considered as a precancerous lesion in gastric cancer (GC). The aim of this study was to identify potential molecules involved in the pathogenesis of CAG in the Tibetan plateau, hoping to help the diagnosis and management of the disease. Atrophic and non-atrophic gastric mucosal tissue samples were collected from seven patients with chronic gastritis (CG). Differentially expressed lncRNAs, circRNAs, miRNAs, and mRNAs between CAG and chronic non-atrophic gastritis (CNAG) groups were identified based on DNBSEQ-G99 RNA sequencing. Subsequently, competitive endogenous RNA (ceRNA) regulatory networks (lncRNA/circRNA-miRNA-mRNA networks) were constructed. Two datasets (GSE153224 and GSE163416), involving data from non-Tibetan plateau areas, were used to further screen out Tibetan plateau key mRNAs, followed by the common genes of Tibetan plateau key and ferroptosis-related mRNAs were also identified. Functional enrichment analyses were performed to investigate the biological functions of Tibetan plateau mRNAs in the CAG. A total of seven lncRNA-miRNA-mRNA relationship pairs and 424 circRNA-miRNA-mRNA relationship pairs were identified in this study. The relationship pairs of hsa_circ_0082984-hsa-miR-204-5p-CACNG8, lncRNA DRAIC/has_circ_0008561-hsa-miR-34a-5p-AR/GXYLT2, lncRNA GAS1RR/RGMB-AS1/hsa_circ_0008561-hsa-miR-3614-5p-TMEM216/SUSD5, and LINC00941/hsa_circ_0082984-hsa-miR-873-3p-TMC5 can be involved in the pathogenesis of CAG. Additionally, eight common genes of Tibetan plateau key and ferroptosis-related differentially expressed mRNAs (DEmRNAs) (CBS, SLC2A4, STAT3, ALOX15B, ATF3, IDO1, NOX4, and SOCS1) were identified in CAG. The common genes of Tibetan plateau key and ferroptosis-related DEmRNAs can play a role in the JAK-STAT signaling pathway. This study identified important molecular biomarkers that may be involved in regulating the pathological mechanisms of CAG in the Tibetan plateau, which provides potential research directions for future research.

## Introduction

Chronic gastritis (CG) manifests as chronic atrophic gastritis (CAG) and chronic non-atrophic gastritis (CNAG)^1^. CNAG may progress to CAG, which is characterized by gastric mucosal atrophy, degeneration intrinsic glands, intestinal metaplasia, and eventually gastric cancer (GC)^2^. As a precancerous lesion of intestinal-type GC, CAG is a digestive system disease characterized by atrophy of the gastric mucosal glands, caused by various pathogenic factors^3,4^. The most common symptoms of CAG include epigastric pain, bloating, abdominal discomfort, and anorexia^5^. Owing to the important role of early diagnosis and treatment in preventing the occurrence of GC, CAG has received more attention in recent years. However, timely diagnosis of CAG remains difficult in clinical practice due to the lack of specificity of clinical symptoms. Currently, CAG is usually diagnosed using endoscopy and pathological examination^6^. However, endoscopic and histological assessments of mucosal atrophy have some limitations, as well as inaccurate localization and misdiagnosis due to inappropriate histopathological examination of section^7^. Therefore, there is an urgent need to identify new diagnostic biomarkers with high sensitivity and specificity for early diagnosis of CAG, with real-time clinical applications.

Tibet is located in the southwestern part of the Qinghai-Tibet Plateau, with an average altitude of over 4000 m, and is known as the third pole in the world^8^. Due to its important geographical and strategic location, medical research in plateau areas has gradually emerged and attracted considerable attention in recent years. In contrast to other areas, most areas in Tibet are high-altitude areas with perennial low oxygen, low pressure, severe cold, relatively poor sanitary conditions, high Helicobacter pylori (mainly east asian type) infection rate^9^, common bile reflux, high salt, fat, and high purine food intake, leading to a high incidence of CAG. However, few studies have been conducted on patients in plateau areas, hindering specific studies on the pathogenesis, early diagnosis, and treatment of CAG in Tibetan plateau areas. Therefore, it is necessary to further explore the molecular development mechanism of CAG in Tibetan plateau areas to provide a theoretical basis for the diagnosis, treatment, and pathogenesis of the disease.

Noncoding RNAs (ncRNAs) are functional RNA molecules that are not translated into proteins, including long noncoding RNAs (lncRNAs) and circular RNAs (circRNAs)^10^, which can act as microRNA (miRNA) sponges, compete with the same corresponding miRNA response element (MRE), and effectively control the subsequent post-transcriptional regulation of miRNA^11^. LncRNAs can act as cis-acting factors to regulate the expression of neighboring genes in the genome, and can also act as trans-acting elements to regulate gene transcription^12^. In addition, lncRNAs may affect the post-transcriptional modification of mRNA. Therefore, lncRNAs are attractive candidates as new diagnostic biomarkers for CAG. Studies have shown that circRNAs have miRNA complementary binding sites and interact with miRNAs to play a regulatory role in diseases^13^, showing great potential to be used as biomarkers and therapeutic targets^14–16^. However, there are few reports on the RNA-mediated regulatory network in CAG, and there are still some deficiencies in the general understanding of this network. Therefore, a systematic understanding of CAG-associated RNA molecular mechanisms is crucial for developing new strategies for early diagnosis and therapeutic intervention of CAG.

**Figure 1:**
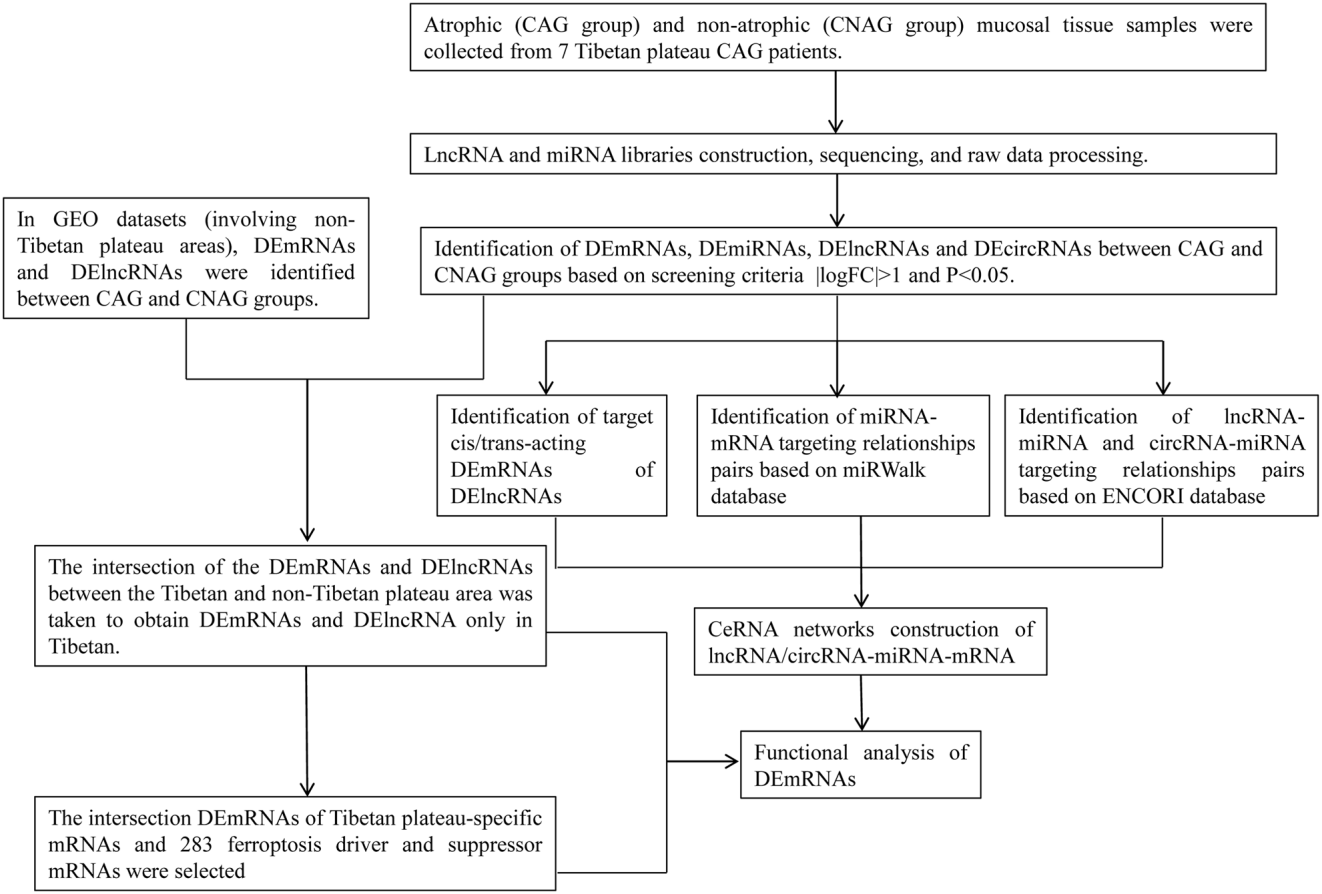
The flowchart of this study.

To explore the molecular development mechanism of CAG in the Tibetan plateau, we collected atrophic and non-atrophic gastric mucosal tissue samples from seven Tibetan patients with CG. Differentially expressed lncRNAs, circRNAs, miRNAs, and mRNAs between CAG and CNAG groups were identified based on DNBSEQ-G99 RNA sequencing. Based on this information, competitive endogenous RNA (ceRNA) regulatory networks (lncRNA/circRNA-miRNA-mRNA network) were developed and GO and KEGG enrichment analyses were performed to detect the biological functions of Tibetan plateau mRNAs. The flowchart of this study is shown in Fig. 1. This study contributes to the identification of potential biomarkers for CAG.

## Materials and methods

### Participants from the Tibetan plateau region

Seven CAG patients from the Tibetan plateau were included in this study. The inclusion criteria to be a participant were as follows: (1) patients belonged to the Tibetan plateau region; (2) patients ranged between 18 and 70 years of age; (3) CAG patients who met the endoscopic diagnosis and pathological diagnosis criteria (the diagnostic criteria are based on the Chinese Consensus on chronic gastritis proposed in 2012); (4) CAG patients who met the determination indicators of pepsinogen, serum gastrin, and immunological examination; and (5) routine endoscopic evaluation of patients with CNAG with lymphocyte infiltration in the gastric mucosa of the gastric antrum. Exclusion criteria for the above individuals were as follows: (1) patients under the age of 18 or over 70 years; (2) patients with gastric ulcer, duodenal ulcer, special type of gastritis, or gastrointestinal bleeding; (3) histopathological examination showing gastric mucosal atrophic changes, dysplasia, or suspicious malignant transformation; (4) patients with a history of gastric surgery; (5) patients with severe heart, lung, liver, kidney, or blood system complications or suffering from life-threatening diseases; (6) patients with a history of mental disorders, or alcohol/drug abuse; (7) female patients who were going to give birth, pregnancy, or breastfeeding. Detailed clinical information of these individuals is showed in Table 1. Atrophic mucosal tissue samples (CAG group) and non-atrophic mucosal tissue samples (CNAG group) from seven CAG patients were collected for further analysis. The atrophic mucosal tissue samples and non-atrophic mucosal tissue samples were obtained from the greater and lesser curvature of the stomach of the same patient, respectively. All participants provided written informed consent. The study was approved by the Ethics Committee of Hospital of Chengdu Office of People’s Government of Tibetan Autonomous Region (Ethics Approval Number: 202137).

**Table 1:** Clinical information registry.

Number	Sex	Age (years)	BMI	Altitude (m)	WBC (10^9/L)	HGB (g/L)	RBC (10^12/L)	PLT (10^9/L)	ALT (U/L)	AST (U/L)	CRE (umol/L)	UREA (umol/L)
CNAG1	Male	62	28.2	3650	6.38	182	5.92	202	38	21	67	3.39
CNAG2	Male	45	23.9	3650	4.94	143	4.78	216	53	23	77	5.75
CNAG3	Male	34	24.6	3600	7.04	184	5.88	232	35	31	80	4.9
CNAG4	Male	59	23.2	3253	5.33	141	4.46	146	19	15	63	3.44
CNAG5	Male	62	26	4350	6.81	160	5.11	130	27	17	75	4.25
CNAG6	Female	53	30.4	4350	6.55	148	5.04	264	28	12	52	5.66
CNAG7	Female	54	24.5	3670	5.75	138	4.49	324	20	14	57	4.51
CAG1	Male	62	28.2	3650	6.38	182	5.92	202	38	21	67	3.39
CAG2	Male	45	23.9	3650	4.94	143	4.78	216	53	23	77	5.75
CAG3	Male	34	25.2	3600	7.04	184	5.88	232	35	31	80	4.9
CAG4	Male	59	23.2	3253	5.33	141	4.46	146	19	15	63	3.44
CAG5	Male	62	26	4350	6.81	160	5.11	130	27	17	75	4.25
CAG6	Female	53	30.4	4350	6.55	148	5.04	264	28	12	52	5.66
CAG7	Female	54	24.5	3670	5.75	138	4.49	324	20	14	57	4.51

Number	Sex	blood sugar (mmol/L)	BUA (mmol/L)	Smoking history	Alcohol history	NSAIDS	Hormone	CCVD	MS	ICD	Samples metaplasia	
CNAG1	Male	6.71	290	Yes	Yes	No	No	No	Yes	No	Intestinal metaplasia	
CNAG2	Male	5.14	387	No	No	No	No	No	No	No	Intestinal metaplasia	
CNAG3	Male	4.69	439	No	No	No	No	No	No	No	Intestinal metaplasia	
CNAG4	Male	5.5	376	Yes	Yes	No	No	No	No	No	Intestinal metaplasia	
CNAG5	Male	4.63	460	Yes	Yes	No	No	No	Yes	No	Intestinal metaplasia	
CNAG6	Female	5.03	236	No	No	No	No	Yes	No	No	No	
CNAG7	Female	4.54	296	No	No	No	No	No	No	No	No	
CAG1	Male	6.71	290	Yes	Yes	No	No	No	Yes	No	Intestinal metaplasia	
CAG2	Male	5.14	387	No	No	No	No	No	No	No	Intestinal metaplasia	
CAG3	Male	4.69	439	No	No	No	No	No	No	No	Intestinal metaplasia	
CAG4	Male	5.5	376	Yes	Yes	No	No	No	No	No	Intestinal metaplasia	
CAG5	Male	4.63	460	Yes	Yes	No	No	No	Yes	No	Intestinal metaplasia	
CAG6	Female	5.03	236	No	No	No	No	Yes	No	No	No	
CAG7	Female	4.54	296	No	No	No	No	No	No	No	No	

*CNAG* chronic non-atrophic gastritis; *CAG* chronic atrophic gastritis; *BMI* body mass index; *WBC* white blood cell; *HGB* hemoglobin; *RBC* red blood cell; *PLT* platelet; *ALT* alanine transaminase; *AST* aspartate transaminase; *CRE* creatinine; *UREA* urea nitrogen; *BUA* blood uric acid; *NSAIDS* Non-steroidal anti-inflammatory drugs; *CCVD* cardiovascular and cerebrovascular disease; *MS* metabolic syndrome; *ICD* Immune complex disease.

### LncRNA library construction, sequencing, and raw data processing

Ribosomal RNA was removed from total RNA using the RNase H method. The purified RNA was fragmented, and one-strand cDNA and two-strand cDNA with DUTP were synthesized in a PCR apparatus. Subsequently, terminal repair and connection of the sequencing connector were performed. The two-stranded cDNA containing the U strand was digested with UDG enzymes and simultaneously subjected to PCR amplification to complete the library preparation. Qualified libraries were sequenced for lncRNA, mRNA and circRNA using PE100 strategy on DNBSEQ-G99 platform. The raw data were quality-controlled using fastp software to obtain high-quality clean data. Specifically, adapter sequence, 5’ segment, 3’ segment, bases with quality < 20 and reads with N > 10% were trimmed. The high-quality clean data of lncRNA and mRNA were aligned to the human reference genome (GRCh38) using the HISAT2 program. In addition, high-quality clean data of circRNA was compared and analyzed using the CIRIquant tool.

### MiRNA library construction, sequencing, and raw data processing

The length of 18–30 nt RNA was recovered from the total RNA. Reverse transcription PCR amplification was performed after addition of adapters at the 3’ and 5’ ends. Subsequently, the cDNA was amplified using highly sensitive polymerase. Library inspection and pooling looping were performed to obtain qualified libraries. Qualified libraries were sequenced for miRNA using SE50 strategy on DNBSEQ-G99 platform. The raw data were quality-controlled using fastp software to obtain high-quality clean data. Specifically, joint sequence, bases with quality < 20 and reads with N > 10% were trimmed. Subsequently, the high-quality clean data after quality control were aligned and annotated using QuickMIRSeq.

### Differential expression analysis and functional annotation

EdgeR package was used to identify differentially expressed lncRNAs (DElncRNAs), differentially expressed miRNAs (DEmiRNAs), and differentially expressed mRNAs (DEmRNAs) between the CAG and CNAG groups. CIRIquant was used to identify differentially expressed circRNAs (DEcircRNAs). DElncRNAs, DEmiRNAs, DEmRNAs, and DEcircRNAs were screened out under the screening criteria of | log fold change (FC) |> 1 and *P* < 0.05. The “pheatmap” and “ggplot2” packages were used to plot the heat maps and volcano maps, respectively. In addition, the clusterProfiler package was used for Kyoto Encyclopedia of Genes and Genomes (KEGG) and Gene Ontology (GO) enrichment analyses of DEmRNAs. KEGG contains numerous signaling pathways^17–19^. Significantly enriched GO and KEGG terms were identified under the screening conditions of *P* < 0.05.

### Identification of target cis/trans-acting DEmRNAs of DElncRNAs

Based on the different modes of action of lncRNAs and target mRNAs, target mRNA prediction was divided into Cis and Trans groups. The mode of action of Cis is to use genome annotation and genome browser to identify possible target mRNAs of lncRNA, which generally refers to mRNA within 100 kb upstream and downstream of lncRNA. Target mRNAs transcribed in the same direction in the promoter region generally promote expression, whereas those transcribed in the opposite direction inhibit expression. |r| represents the correlation coefficient between lncRNA and mRNA, which is used to measure the degree of linear correlation between lncRNA and mRNA expression levels in the same sample. A positive value of r indicates a positive correlation, while a negative value of r indicates a negative correlation. The screening criteria for target cis-acting mRNAs of lncRNAs were |r|> 0.8 and *P* < 0.05. |r|> 0.8 indicates that only lncRNA-mRNA pairs with absolute correlation coefficients greater than 0.8 are selected as candidate relationship pairs for cis-acting. This standard indicates a strong linear correlation. The trans action mode suggests that the function of lncRNA is not only related to the position of the coding mRNA but is also related to protein-coding genes co-expressed with lncRNA. The screening criteria for target trans-acting mRNAs of lncRNAs were |r|> 0.95 and *P* < 0.05. |r|> 0.95 indicates that only lncRNA-mRNA pairs with absolute correlation coefficients greater than 0.95 are selected as candidate relationship pairs for trans-acting. This standard indicates a strong linear correlation. The lncRNA-mRNA regulatory network was visualized using Cytoscape.

### CeRNA network construction of lncRNA/circRNA-miRNA-mRNA

The miRWalk database was used to identify miRNA-mRNA targeting relationships and screen out experimentally validated targeting relationship pairs. The ENCORI database was used to identify the target pairs of lncRNA-miRNA and circRNA-miRNA. CeRNA network of lncRNA/circRNA-miRNA-mRNA were constructed after the fusion of different relationship pairs.

### Further difference analyses of molecules in Tibetan plateau areas based on online dataset (involved non-Tibetan plateau areas)

Two datasets, including data from non-Tibetan plateau areas, were downloaded from the Gene Expression Omnibus (GEO) database: the GSE153224 dataset (Changchun area) and GSE163416 dataset (Wuxi area). In addition, the data were standardized and normalized. The edgeR package was used to analyze the DEmRNAs and DElncRNAs of CAG patients using the screening criteria of |logFC|> 1 and *P* < 0.05. The intersection of the DEmRNAs and DElncRNAs between the Tibetan and non-Tibetan plateau area was taken to obtain Tibetan plateau key DEmRNAs. The Venn in R package was used to create a Venn diagram. In addition, 283 ferroptosis driver and suppressor mRNAs were identified in a previous report^20^. The common genes of Tibetan plateau key and ferroptosis-related DEmRNAs were further identified in patients with CAG.

## Results

### Screening of DElncRNAs, DEcircRNAs, DEmiRNAs, and DEmRNAs in Tibetan plateau areas

**Figure 2:**
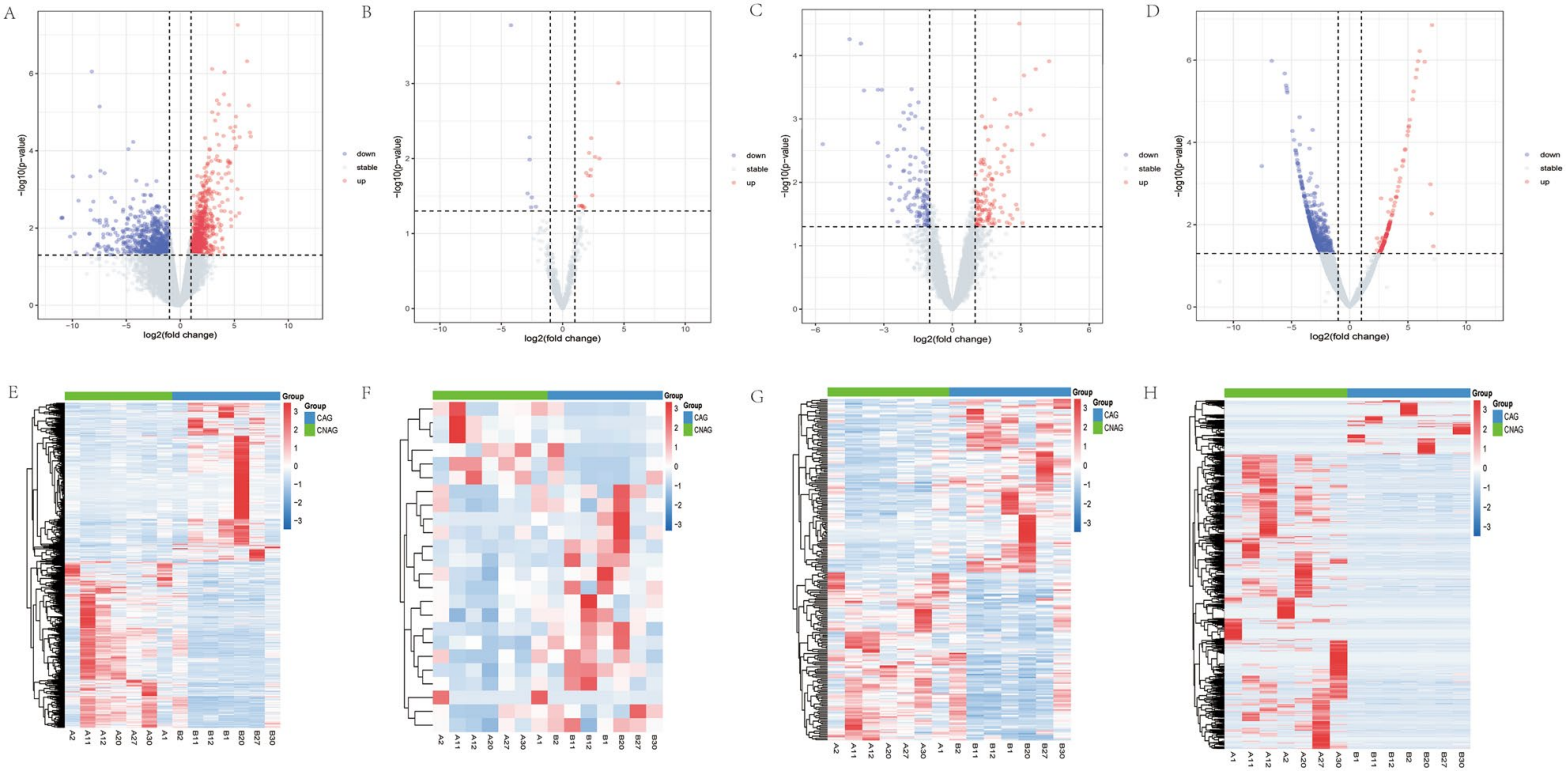
Volcano plots and heatmaps of DEmRNAs, DEmiRNAs, DElncRNAs, and DEcircRNAs between CAG and CNAG groups. (**A**–**D**) The volcano plots of DEmRNAs/DEmiRNAs/DElncRNAs/DEcircRNAs. Red dots represents up-regulated in the CAG group. Blue dots represents down-regulated in the CAG group. (**E**–**H**) The heatmaps of DEmRNAs/DEmiRNAs/DElncRNAs/DEcircRNAs. Red represents up-regulated, blue represents down-regulated. *CAG* chronic atrophic gastritis; *CNAG* chronic non-atrophic gastritis.

A total of 750 DEmRNAs, 24 DEmiRNAs, 242 DElncRNAs, and 1353 DEcircRNAs were identified between CAG and CNAG groups. Volcano and heatmaps of these molecules are shown in Fig. 2.

### Target mRNAs prediction of lncRNAs in Tibetan plateau areas

**Figure 3:**
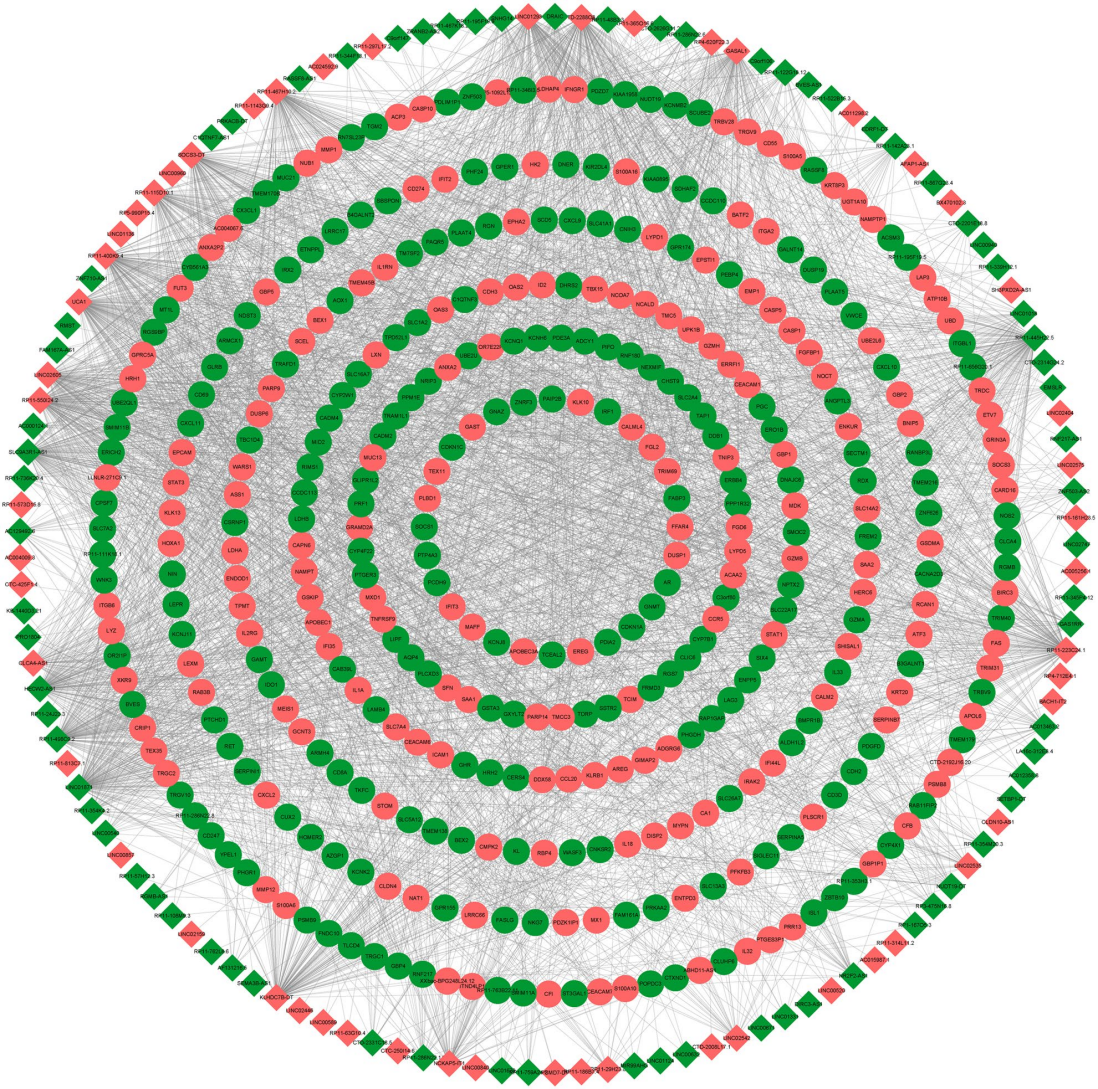
Visualization of lncRNA target mRNAs prediction in CAG and CNAG groups. Pink represents up-regulated, green represents down-regulated. Circles represents mRNAs, rhombuses represents lncRNAs. *CAG* chronic atrophic gastritis; *CNAG* chronic non-atrophic gastritis.

According to the screening criteria of |r|> 0.8 and *P* < 0.05, 15 cis-acting lncRNA-mRNA relationship pairs (including 25 mRNAs) were identified. In addition, according to the screening criteria of |r|> 0.95 and *p* value < 0.05, 3028 trans-acting lncRNA-mRNA relationship pairs (including 390 mRNAs) were identified. The lncRNA-mRNA regulatory network is shown in Fig. 3, which includes 3049 lncRNA-mRNA relationship pairs and 397 mRNAs. In the lncRNA-mRNA regulatory network, pink, green, circles and rhombuses represent up-regulated, down-regulated, mRNAs and lncRNAs, respectively.

### CeRNA networks construction of lncRNA/circRNA-miRNA-mRNA in Tibetan plateau areas

**Figure 4:**
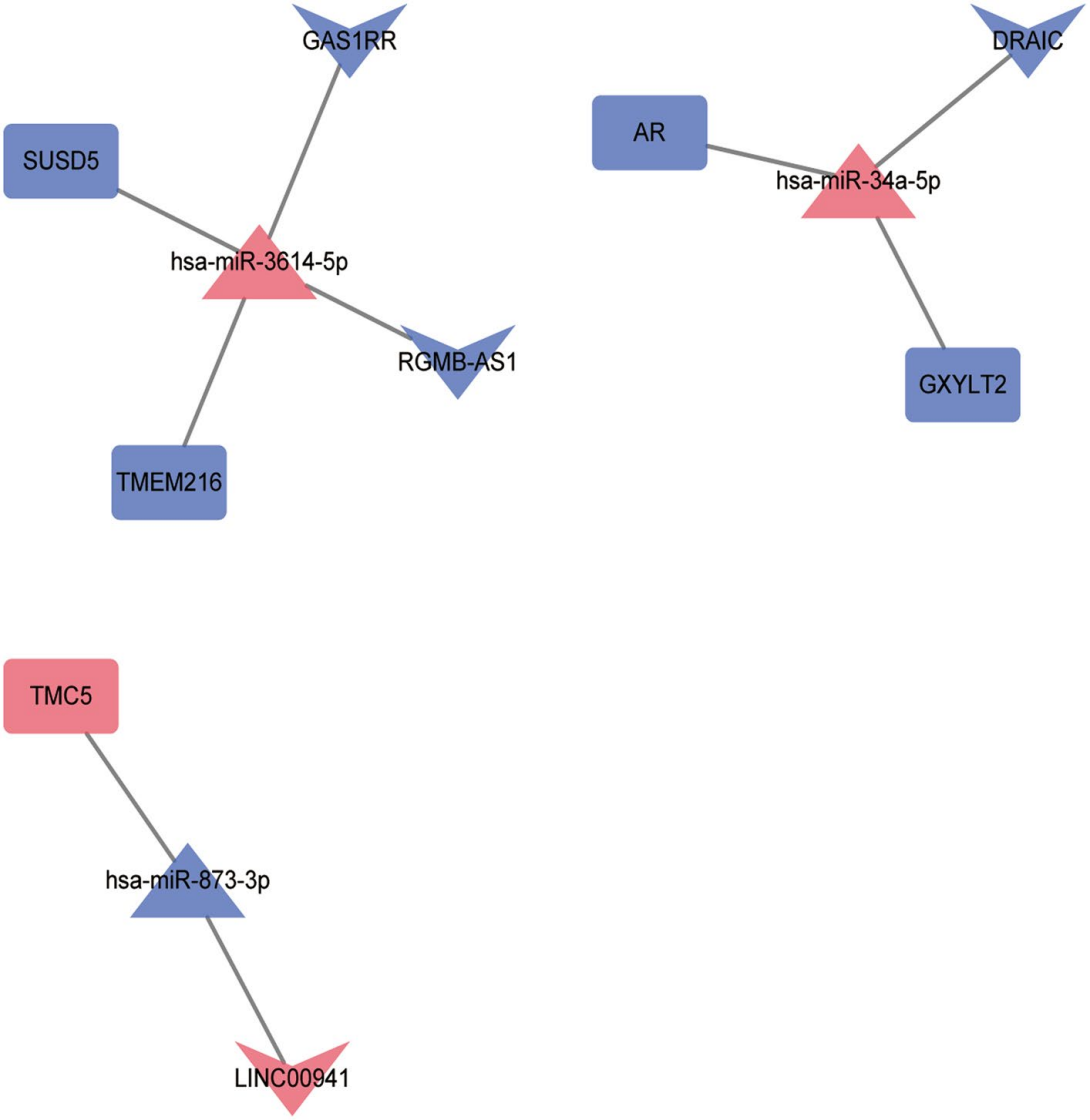
The ceRNA network of lncRNA-miRNA-mRNA in CAG and CNAG groups. Pink represents up-regulated, blue represents down-regulated. Rectangles represents mRNAs, triangles represents miRNAs, V-shaped represents lncRNAs. *CAG* chronic atrophic gastritis; *CNAG* chronic non-atrophic gastritis.

The ceRNA (lncRNA/circRNA-miRNA-mRNA) network was developed by integrating lncRNA/circRNA-miRNA and miRNA-mRNA relationship pairs. Seven lncRNA-miRNA-mRNA (involving five mRNAs) relationship pairs (LINC00941-hsa-miR-873-3p-TMC5, GAS1RR-hsa-miR-3614-5p-TMEM216, GAS1RR-hsa-miR-3614-5p-SUSD5, RGMB-AS1-hsa-miR-3614-5p-TMEM216, RGMB-AS1-hsa-miR-3614-5p-SUSD5, DRAIC-hsa-miR-34a-5p-AR and DRAIC-hsa-miR-34a-5p-GXYLT2) were identified in lncRNA-miRNA-mRNA network (Fig. 4). In the lncRNA-miRNA-mRNA network, pink, blue, rectangles, triangles and V-shaped represent up-regulated, down-regulated, mRNAs, miRNAs and lncRNAs, respectively. LINC00941-hsa-miR-873-3p-TMC5, GAS1RR/RGMB-AS1-hsa-miR-3614-5p-TMEM216/SUSD5, and DRAIC-hsa-miR-34a-5p-AR/GXYLT2 could be involved in the occurrence and development of CAG in Tibetan plateau areas.

**Figure 5:**
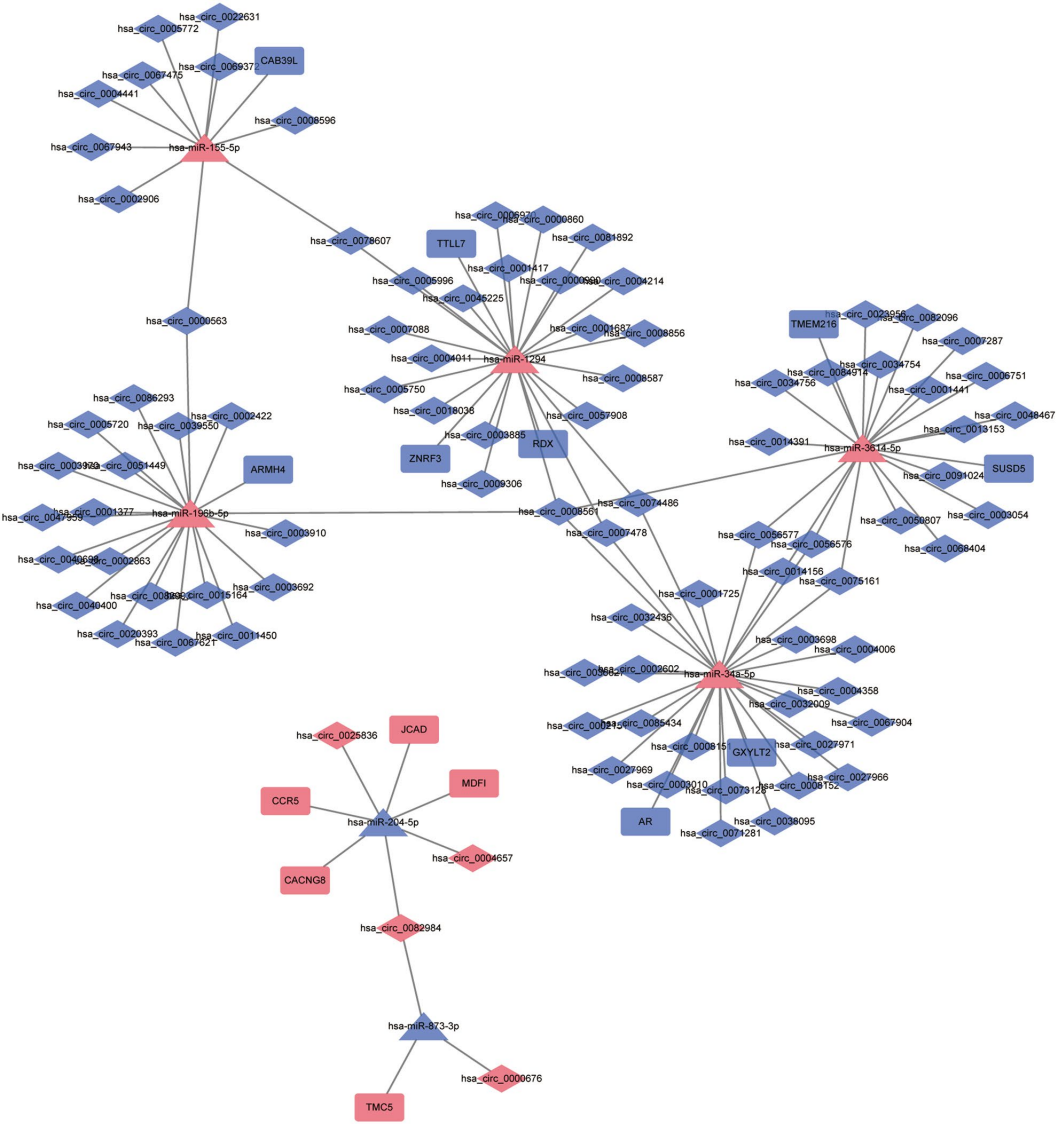
The ceRNA network of circRNA-miRNA-mRNA in CAG and CNAG groups. Pink represents up-regulated, blue represents down-regulated. Rectangles represents mRNAs, triangles miRNAs, rhombuses represents circRNAs. *CAG* chronic atrophic gastritis; *CNAG* chronic non-atrophic gastritis.

In addition, 204 circRNA-miRNA-mRNA (involving 14 mRNAs) relationship pairs were identified in circRNA-miRNA-mRNA network (Fig. 5). In the circRNA-miRNA-mRNA network, pink, blue, rectangles, triangles and rhombuses represent up-regulated, down-regulated, mRNAs, miRNAs and circRNAs, respectively. Among which, hsa_circ_0025836/hsa_circ_0082984/hsa_circ_0004657-hsa-miR-204-5p-CACNG8/JCAD/CCR5/MDFI, hsa_circ_0074486/hsa_circ_0008561-hsa-miR-34a-AR/GXYLT2, hsa_circ_0008561-hsa-miR-3614-5p-TMEM216/SUSD5, hsa_circ_0074486/hsa_circ_0008561-hsa-miR-129-RDX, hsa_circ_0082984/hsa_circ_0000676-hsa-miR-873-3p-TMC5 may be involved in the pathogenesis of CAG.

### Identification of the common genes of Tibetan plateau key and ferroptosis related DEmRNAs in CAG patients based on online dataset

**Figure 6:**
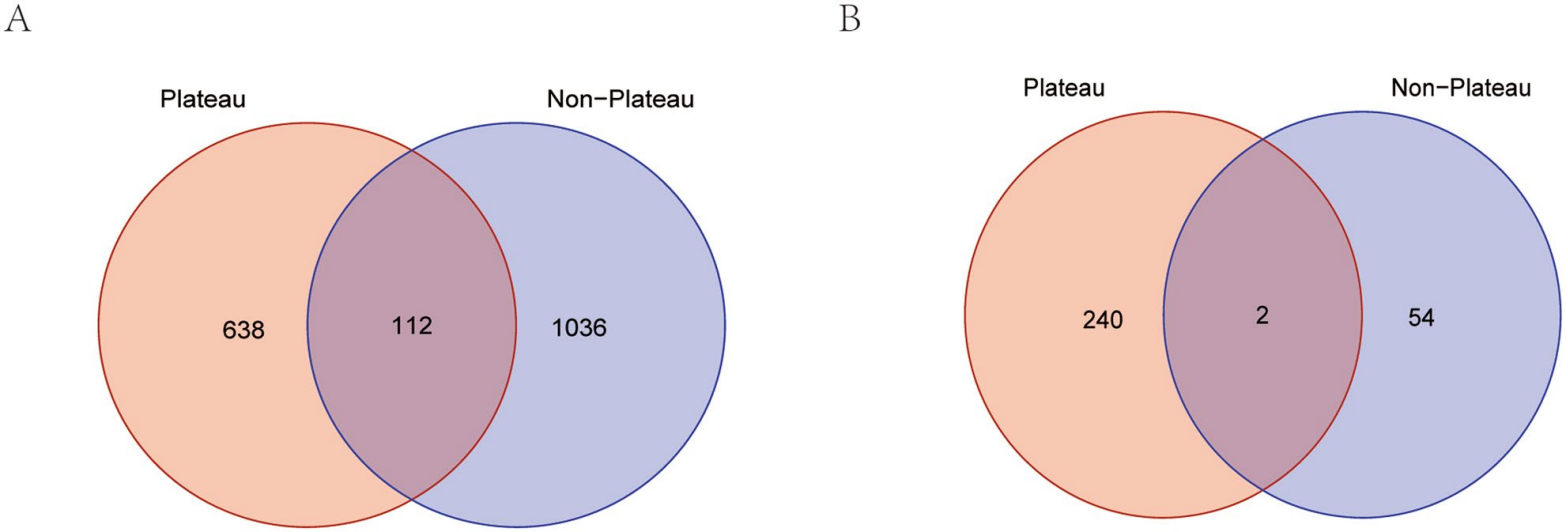
Venn diagrams of DEmRNAs (**A**) and DElncRNAs (**B**) between Tibetan plateau areas and non-Tibetan plateau areas in CAG and CNAG groups. *CAG* chronic atrophic gastritis; *CNAG* chronic non-atrophic gastritis.

In the GEO dataset (involving non-Tibetan plateau areas), a total of 1148 DEmRNAs and 56 DElncRNAs were identified between CAG and CNAG groups. A total of 112 common DEmRNAs, 2 common DElncRNAs, 638 Tibetan plateau key DEmRNAs, and 240 Tibetan plateau key DElncRNAs were obtained by intersection processing of DEmRNAs and DElncRNAs from Tibetan plateau and non-Tibetan plateau areas (Fig. 6A,B). It is noted that eight common DEmRNAs were further identified after taking the intersection of 283 ferroptosis genes and 638 Tibetan plateau key mRNAs, including CBS, SLC2A4, STAT3, ALOX15B, ATF3, IDO1, NOX4, and SOCS1.

### Functional analysis of DEmRNAs in CAG patients in Tibetan plateau areas

**Figure 7:**
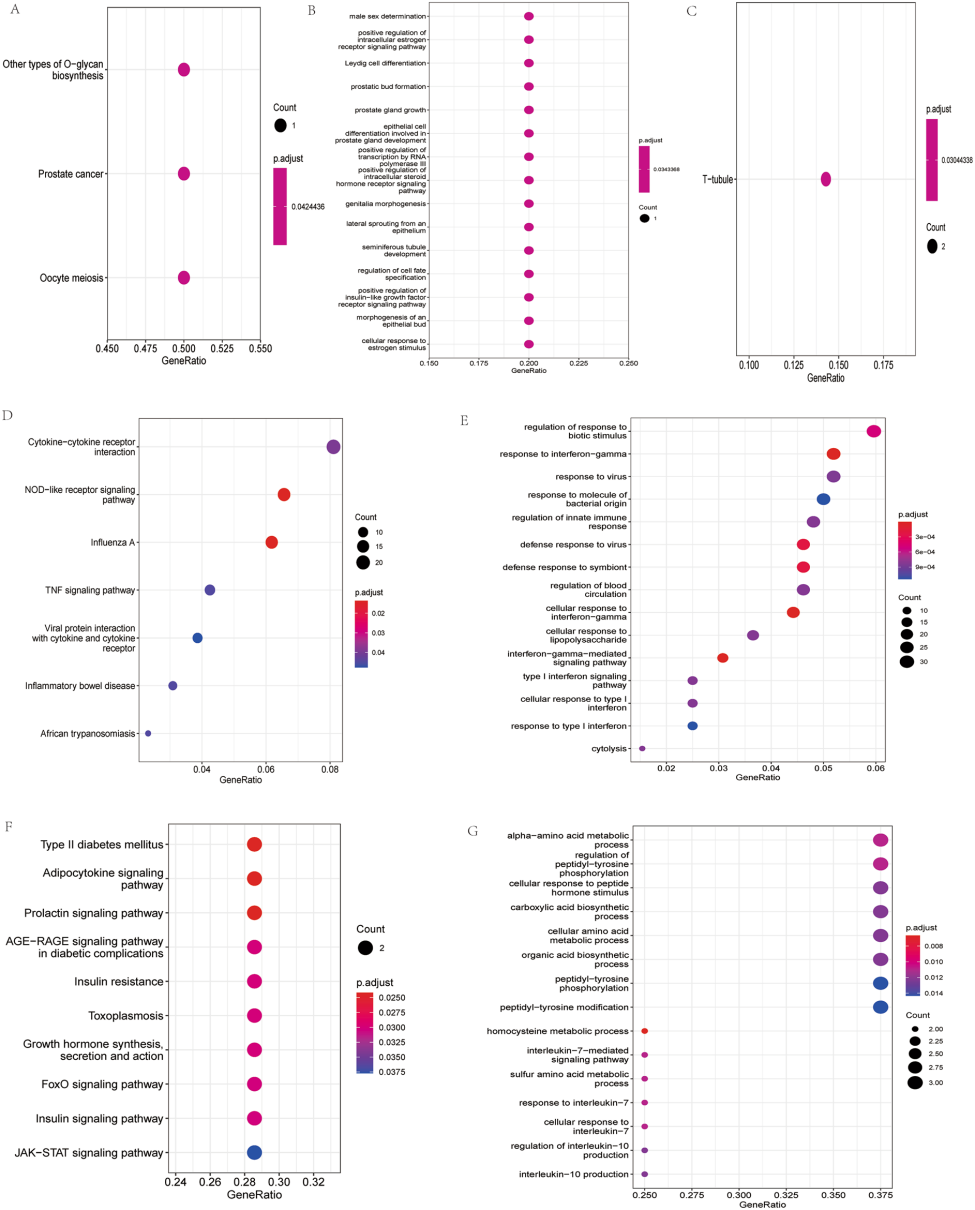
KEGG and GO enrichment analysis of DEmRNAs between CAG and CNAG groups in the Tibetan plateau areas. (**A**, **B**) KEGG and GO enrichment analysis of DEmRNAs in mRNA-miRNA-lncRNA network; (**C**) GO enrichment analysis of DEmRNAs in mRNA-miRNA-circRNA network; (**D**, **E**) KEGG and GO enrichment analysis of Tibetan plateau key DEmRNAs; (**F**, **G**) KEGG and GO enrichment analysis of common genes of Tibetan plateau key and ferroptosis related DEmRNAs. *CAG* chronic atrophic gastritis; *CNAG* chronic non-atrophic gastritis; *KEGG* Kyoto encyclopedia of genes and genomes; *GO* gene ontology.

To understand the mechanisms of DEmRNAs involved in CAG in Tibetan plateau areas, GO enrichment and KEGG pathway analyses were performed. KEGG analysis of mRNAs in the lncRNA-miRNA-mRNA regulatory network showed that GXYLT2 was enriched in other types of O-glycan biosynthesis, and AR was enriched in prostate cancer and oocyte meiosis pathways (Fig. 7A). AR, TMEM216, GXYLT2, TMC5, and SUSD5 were involved in some GO terms (Fig. 7B). GO analysis of mRNAs in the circRNA-miRNA-mRNA regulatory network showed that RDX and CACNG8 were enriched in the T-tubules in terms of cellular components (Fig. 7C).

Functional analysis of 638 Tibetan plateau key DEmRNAs was also performed. These mRNAs were mainly enriched in the NOD-like receptor signaling pathway, cytokine–cytokine receptor interaction KEGG pathways (Fig. 7D). These mRNAs also participated in some biological processes, such as regulation of response to biotic stimuli, regulation of blood circulation, and type I interferon signaling pathway (Fig. 7E). KEGG pathways of Tibetan plateau key and ferroptosis-related DEmRNAs are shown in Fig. 7F. STAT3/SOCS1 is involved in the JAK-STAT signaling pathway. SLC2A4/STAT3 is involved in the adipocytokine and FoxO signaling pathways. Some enriched biological processes were identified, such as alpha-amino acid metabolic processes and the regulation of peptidyl-tyrosine phosphorylation (Fig. 7G).

## Discussion

CAG is a digestive system disease, which is a precancerous lesion state of GC^21,22^. Zhang et al. showed that the incidence of CG in plateau residents was higher than that in plain residents, and CAG was more common, which increased with increasing altitude^23^. However, there has been little exploration of patients in the Tibetan plateau areas in recent years. Therefore, exploring the molecular mechanism of CAG in Tibetan plateau areas can provide a theoretical basis for the diagnosis, treatment, and pathogenesis of the disease. In recent years, ceRNA regulatory networks have played an important role in disease development and have been used in disease diagnosis, treatment, and prognosis prediction. CircRNAs and lncRNAs can be associated with multiple mRNAs and miRNAs. Based on the differential expression of lncRNAs, circRNAs, miRNAs, and mRNAs in CAG patients in Tibetan plateau areas, ceRNA regulatory networks (lncRNA/circRNA-miRNA-mRNA network) in CAG were constructed in this study. Moreover, Tibetan plateau key DEmRNAs and lncRNAs were also identified. It is noted that eight common DEmRNAs were further identified after taking the intersection of 283 ferroptosis genes and 638 Tibetan plateau key mRNAs. In addition, biological pathways that may be involved in CAG in Tibetan plateau areas were identified through functional analysis.

The lncRNA DRAIC is significantly correlated with the clinicopathological features of immune cell infiltration, tumor stage, and lymph node metastasis^24^, and its expression is down-regulated in GC tissues and cell lines, functioning as a tumor suppressor^25^. LncRNA GAS1RR is down-regulated in prostate cancer (PCa) tissues^26^. RGMB-AS1 is up-regulated in GC cells and promotes GC progression by regulating the miR-22-3p/NFIB axis^27^. LINC00941 is highly expressed in GC tissues^28^ and plays an important oncogenic role in GC^29^. Has-miR-204-5p has been shown to inhibit lipopolysaccharide (LPS)-induced inflammatory processes in microglia^30^, and miR-204-5p can suppress the IL-2-mediated inflammatory response in HK-6 renal tubular epithelial cells^31^. Hsa-miR-34a-5p is involved in the regulation of TLR signaling and NF-κB-mediated inflammatory responses^32^ and plays a crucial role in the occurrence of inflammation and autoimmune diseases^33^. Down-regulated of has-miR-34a-5p was found in inflammatory factor-stimulated bronchial epithelial cell-derived exosomes^34^, which was consistent with the expression of has-miR-34a-5p in CAG in our study. In addition, hsa-miR-34a-5p is a p53-regulated tumor suppressor involved in the progression of various cancers^35^. Fang et al.^36^ confirmed that miR-873-3p is a target gene of LINC00941, which is down-regulated in pancreatic adenocarcinoma (PAAD) tissues. In summary, miRNAs mediate various inflammatory responses; most lncRNAs are reported to be related to cancer, and their expression in CAG is a novel finding of our study.

CACNG8 is preferentially expressed in the hippocampus, cortex, and subcortical regions, which is critical for mood production, suggesting a relationship with psychiatric disorders^37^. However, high expression of CACNG8 in CAG tissues was found for the first time in our study. We speculate that mood plays an important role in CAG development. AR shares high homology with GR and is a steroid hormone receptor with potent anti-inflammatory properties^38^. Disruption of GR signaling can induce spontaneous gastric inflammation and chemosis in female mice^39^. GXYLT2 is considered an important gene that regulates canonical Notch signaling, participates in human tumor progression, and is closely related to tumor immune infiltrating cells and immune genes^40,41^. Zhang et al.^42^ showed that high levels of TMC5 are activators of PI3K/AKT, RAS/MAPK, and TSC/mTOR, and it has been reported that activation of these pathways may lead to dysregulation of cell survival and ultimately tumorigenesis^43^ and various cancers^44^. These genes are involved in the process of inflammation and immune regulation and play an important role in the progression of CAG. In this study, we identified regulatory relationship pairs of hsa_circ_0082984-hsa-miR-204-5p-CACNG8, lncRNA DRAIC/has_circ_0008561-hsa-miR-34a-5p-AR/GXYLT2, lncRNA GAS1RR/RGMB-AS1/hsa_circ_0008561-hsa-miR-3614-5p-TMEM216/SUSD5, and LINC00941/hsa_circ_0082984-hsa-miR-873-3p-TMC5 that may play an important role in CAG.

In addition, through the screening of differential genes between the Tibetan and non-Tibetan plateau areas, it was further proved that patients with CAG in Tibetan plateau areas had unique mRNA and lncRNA expression patterns. The KEGG pathway of common genes of Tibetan plateau key and ferroptosis-related DEmRNAs showed that STAT3/SOCS1 was involved in the JAK/STAT pathway, and SLC2A4/STAT3 was involved in the adipocytokine and FoxO signaling pathways. Helicobacter pylori infection induces many pro-inflammatory signaling pathways, leading to gastric inflammation and carcinogenesis, and is one of the early response pathways to Helicobacter pylori infection, which mediates immune regulatory processes and contributes to immune escape and the development of GC^45^. The FOXO signaling pathway is involved in oxidative stress and inflammation in Parkinson’s^46^. Eating disorders and abnormal body weight may be associated with dysregulation of the adipocytokine signaling pathway^47^, which may be related to the occurrence of gastric diseases. Enrichment analysis of the common genes of Tibetan plateau key and ferroptosis-related DEmRNAs further indicated that the exploration of markers in patients with CAG is a meaningful study.

However, the present study also has a certain degree of limitation. Firstly, the sample size of the sequencing data was small, and it is necessary to continue to expand the sample for further validation. Secondly, the specific molecular mechanisms of the identified lncRNA, circRNA, miRNA and mRNA were still unclear and need to be investigated by a large number of in vitro experiments in the future.

## Conclusions

In summary, some pairs of lncRNA/circRNA-miRNA-mRNA regulatory networks may be involved in the occurrence and development of CAG. The JAK-STAT, adipocytokine, and FoxO signaling pathways are closely related to the occurrence of CAG. This study is helpful for exploring the mechanism of the occurrence and development of CAG in Tibetan plateau areas.

## Data Availability

The datasets presented in this study can be found in online repositories. The name of the repository and accession number can be found below: Gene Expression Omnibus (https://www.ncbi.nlm.nih.gov/geo/query/acc.cgi?acc=GSE229902); GSE229902. GSE15322 dataset (https://www.ncbi.nlm.nih.gov/geo/query/acc.cgi?acc=GSE153224) and GSE163416 (https://www.ncbi.nlm.nih.gov/geo/query/acc.cgi?acc=GSE163416) dataset were downloaded from the GEO database (https://www.ncbi.nlm.nih.gov/geo/).
